# Martin’s Elastic Bandage

**Published:** 1880-07

**Authors:** 


MARTIN'S ELASTIC BANDAGE.—We are glad to see evidence of increasing appreciation of the adaptability and usefulness of the elastic bandage, in the hands of several prominent members of the profession. The field of its usefulness is a large one, and we expect to see its application, or at least the *principle* upon which its efficiency depends, very much extended ere long. And when such is the case we will be better enabled to appreciate a most important and almost *indestructible element*, that is innate in muscular fibre, viz: contractility; inherent in it not only in life, but extending beyond the culinary process, and down even to the confines of disintegration; and is only wholly lost in decomposition! It is therefore of the greatest consequence that it should be recognized and its importance duly appreciated whenever appliances of whatever character are to be made to the skin, whether for momentary or continuous action upon it, or on the subjacent tissues or organs!

We have endeavored for many years to estimate, as nearly as we could approximate the value of this important factor, in our diagnostic, prognostic and therapeutic deductions, and conclusions, at the bedside; and are more and more fully persuaded every day, that the oftener it is regarded in our ministrations to our patients, the greater will be our success in the management of their cases. Most of the local appliances loose a certain per centage of their force, more or less early after being brought in contact with the surface or skin, by the gradual shrinking thereof, from pressure chiefly, and other minor causes, such as the stretching of adhesive strips, bandages, etc., and therefore require early renewal or re-application, in order to the proper carrying out of the object calling for their use originally. The difficulties, and often injury, resulting from such early re-adjustings, and re-newals of bandages, etc., are completely overcome by the use of the elastic bandage, which contracts *pari passu* with the shrinkage or reduction taking place in the limb, or parts, to which it may be applied.

The idea of using a *gum elastic bandage*, (suspender,) was first utilized in this way, so far as we know, in a case of hydrocephalus, that was treated by us, and published in the *Charleston Medical Journal and Review*, volume 7, 1852, page 774, and not volume 8, as stated by Professor Henry H. Smith, M. D., in the second edition, page 60. of his valuable work, "A System of Operative Surgery," Since that case we have never lost sight of the value of elastic compression in whitlow, enlarged joints, in support of the abdomen, in the latter period of gestation, etc., and when we read an account of the use of Martin's elastic bandage a short while ago, we were prepared to endorse *all* the advantages claimed for it as a surgical appliance. This subject may be reverted to again.

